# Solution structure of human P1•P2 heterodimer provides insights into the role of eukaryotic stalk in recruiting the ribosome-inactivating protein trichosanthin to the ribosome

**DOI:** 10.1093/nar/gkt636

**Published:** 2013-07-26

**Authors:** Ka-Ming Lee, Kazuyuki Yusa, Lai-On Chu, Conny Wing-Heng Yu, Moe Oono, Tomohiro Miyoshi, Kosuke Ito, Pang-Chui Shaw, Kam-Bo Wong, Toshio Uchiumi

**Affiliations:** ^1^School of Life Sciences, Centre for Protein Science and Crystallography, The Chinese University of Hong Kong, Shatin, Hong Kong, China and ^2^Department of Biology, Faculty of Science, Niigata University, Ikarashi 2-8050, Nishi-ku, Niigata 950-2181, Japan

## Abstract

Lateral ribosomal stalk is responsible for binding and recruiting translation factors during protein synthesis. The eukaryotic stalk consists of one P0 protein with two copies of P1•P2 heterodimers to form a P0(P1•P2)_2_ pentameric P-complex. Here, we have solved the structure of full-length P1•P2 by nuclear magnetic resonance spectroscopy. P1 and P2 dimerize via their helical N-terminal domains, whereas the C-terminal tails of P1•P2 are unstructured and can extend up to ∼125 Å away from the dimerization domains. ^15^N relaxation study reveals that the C-terminal tails are flexible, having a much faster internal mobility than the N-terminal domains. Replacement of prokaryotic L10(L7/L12)_4_/L11 by eukaryotic P0(P1•P2)_2_/eL12 rendered *Escherichia coli* ribosome, which is insensitive to trichosanthin (TCS), susceptible to depurination by TCS and the C-terminal tail was found to be responsible for this depurination. Truncation and insertion studies showed that depurination of hybrid ribosome is dependent on the length of the proline-alanine rich hinge region within the C-terminal tail. All together, we propose a model that recruitment of TCS to the sarcin-ricin loop required the flexible C-terminal tail, and the proline-alanine rich hinge region lengthens this C-terminal tail, allowing the tail to sweep around the ribosome to recruit TCS.

## INTRODUCTION

The ribosomal stalk of the large subunit of ribosome is responsible for domain-specific actions of translation factors (1) in protein synthesis among the three domains of life. Among bacteria, archaea and eukaryotes, their ribosomal stalk share similar features. They all have an anchorage protein (L10 in bacteria, and P0 in archaea and eukaryotes) whose N-terminal domain directly binds to the ribosomal RNA. From the RNA-binding domain, a spine helix is protruding out for binding dimers of acidic ribosomal proteins, so-called the stalk dimers. In bacteria, two or three L7/L12 homodimers bind to the spine helix of L10 (2,3), whereas three P1 homodimer bind to spine helix of P0 in archaea (4). The situation is different and more complex in eukaryotes, which involve two proteins P1 and P2, forming heterodimers. Two P1•P2 heterodimers was found to bind the spine helix of P0 and specific orientation of these heterodimers on the spine helix was proposed (5–8).

Besides sitting on the ribosome, P1 and P2 were also found free in the cytoplasm and exchange with those on ribosomes (9). P2 forms homodimer in solution (10–12). In the absence of P2, P1 is rapidly degraded in yeast (13). Suppression of P2 expression by RNA interference in human cell lines also leads to the depletion of P1 protein (14). We also showed that the formation of P1•P2 heterodimer is a spontaneous process in which the less stable P2 homodimer is displaced by P1 to form a more stable P1•P2 heterodimer (7). Therefore, these observations indicated that P1 is protected from degradation by forming a more stable P1•P2 complex. Truncation study showed that this stable P1•P2 heterodimer is formed via their N-terminal domain (15), and the structure of this dimerization domain was recently solved (8). This structure not only provides insight on how P1•P2 anchor on the spine helix during stalk assembly, but modeling of this heterodimer on the ribosome also predicted how the C-terminal tails of P1•P2 sweep around the ribosome (8). There is a conserved motif SDDDMGFGLFD at the C-termini of P1, P2 and P0 (called P-proteins for the three proteins), which is involved in binding of ribosomal inactivating proteins, like trichosanthin (TCS) (16,17), ricin-A-chain (18), Shiga-like toxin 1 (19) and maize ribosome-inactivating protein (20). On the other hand, Pokeweed antiviral protein (PAP) was found to interact with ribosomal protein L3 (21). Interaction with ribosomal protein was thought to be important for the inactivating activity as ricin-A-chain cleaves naked RNA 10^5^ times slower than RNA in intact rat ribosome (22). To complete the story on how TCS is recruited by P1•P2 heterodimer and carries out its inactivating activity, we have determined the structure of full-length P1•P2 heterodimer and shows that P1•P2 has a helical N-terminal domain and unstructured C-terminal tails. ^15^N relaxation study reveals that N-terminal dimerization domain and the C-terminal tails have different dynamic properties, with C-terminal tail having much faster internal mobility. The C-terminal tails were found to be required for the depurination activity of TCS, and the level of depurination depends on the length of the proline-alanine rich hinge region within the C-terminal tail. Finally, a structural model on how TCS is recruited by the stalk complex to the sarcin-ricin loop was proposed.

## MATERIALS AND METHODS

### Plasmid construction

Coding DNA sequences of human (*Homo sapiens*) ribosomal stalk proteins HsP1 and HsP2 were amplified by PCR and cloned into pET8c expression vector. The truncation mutants lacking the C-terminal 45 amino acids of HsP1 (HsP1_ΔC_) and lacking 46 amino acids of HsP2 (HsP2_ΔC_) were constructed as described previously (7). The plasmid containing the gene of maltose-binding protein (MBP) fused with C-terminal 36 amino acids of HsP2 was as described (16). The plasmids for the expression of silkworm (*Bombyx mori*) BmP1, BmP2, BmP0 and BmL12 were as described (5). The plasmids for (i) BmP1_ΔH_ and (ii) BmP2_ΔH_, which lacked most parts of the hinge regions in BmP1 (residues 65–87) and BmP2 (residues 59–85), respectively (see Figure 5A), (iii) BmP1_SH_ and (iv) BmP2_SH_, in which six consecutive alanine residues were truncated from the hinge regions of P1 (residues 78–83) and P2 (residues74–79), respectively, (v) BmP1_LH_ and (vi) BmP2_LH_, in which additional six consecutive alanine residues were inserted into the hinge regions of P1 (between residues 80 and 81) and P2 (between residues 76 and 77), respectively, were constructed by inverse PCR using plasmids containing P1 or P2 gene as a template and primers shown in Supplementary Table S1. The C-terminal truncation mutants lacking 52 amino acids of BmP1 (BmP1_ΔC_), 50 amino acids of BmP2 (BmP2_ΔC_) and 55 amino acids of BmP0 (BmP0_ΔC_) were constructed as described previously (5,15).

### Sample preparations

*Asymmetrically labeled P1•P2 heterodimer for nuclear magnetic resonance experiments.* ^13^C-^15^N labeled HsP1 and HsP2 were expressed in *Escherichia coli* strain C41(DE3) in M9 medium (6 g/l Na_2_HPO_4_, 3 g/l KH_2_PO_4_, 0.5 g/l NaCl, 2 mM MgSO_4_) containing 2 g/l ^13^C glucose, 1 g/l ^15^N ammonium chloride and 100 µg/ml ampicllin. To purify HsP1, cell pellet (from 1 l of culture) was resuspended with 30 ml of 20 mM 2-mercaptoethanol, 20 mM Tris–HCl (pH 7.8) (buffer A) and lysed by sonication. The filtered supernatant of the cell lysate was loaded to an Econo column (Bio-rad) containing ∼20 ml of Q sepharose fast flow resin (GE Healthcare). Resin was mixed thoroughly with the supernatant and then incubated at room temperature for 15 min. Then flow-through was collected. The flow-through was subject to 40% ammonium sulfate precipitation at 4°C with gentle stirring for 30 min. Then, the precipitate was pelleted by centrifugation at 10 000*g* for 15 min at 4°C. The pellet was then completely dissolved in 150 ml of buffer A containing 8 M urea (denaturing buffer A) and loaded to 5 ml of HiTrap Q HP column (GE Healthcare) equilibrated with denaturing buffer A. After extensive washing with denaturing buffer A, a gradient of 200 ml from 0 to 0.5 M NaCl was used to elute HsP1. HsP1 was eluted at ∼0.2 M NaCl. To obtain folded HsP1, urea-denatured HsP1 was dialysed against 2 l of 0.2 M NaCl, 20 mM 2-mercaptoethanol, 20 mM Tris–HCl (pH 7.8), twice at 4°C. Purification of HsP2 was described previously (16). To purify HsP1**•**HsP2 heterodimer, excess HsP1 was mixed with HsP2 in molar ratio 2:1 and incubated at 4°C overnight. The protein mixture was then concentrated to <5 ml and loaded to HiLoad Superdex 200 gel filtration column (GE Healthcare). Excess HsP1 formed soluble aggregate and eluted at void volume, whereas HsP1**•**HsP2 heterodimer was eluted at ∼200 ml. Protein was concentrated to 1 mM for nuclear magnetic resonance (NMR) experiements.

*Ribosomal proteins and their variants for functional assays.* Human HsP1_ΔC_•HsP2_ΔC_ heterodimer was prepared as described previously (8). MBP fused with the C-terminal 36 amino acids of HsP2 (MBP-C36) was overexpressed and purified as described (16). Silkworm ribosomal proteins BmP1, BmP2, BmP0, BmL12 (equivalent to *E. coli* L11), and their variants were overexpressed in *E. coli* and purified as described previously (5). The BmP0•BmP1•BmP2 stalk complexes were reconstituted by mixing isolated BmP0, BmP1 and BmP2, or alternative individual variants, in the presence of 7 M urea, and by removing gradually urea, as described previously (23). The *E. coli* stalk complex L10•L7/L12 and L11 were prepared, as described previously (24).

*Ribosomes and hybrid ribosomes*. The eukaryotic 80 S ribosomes were prepared from *Artemia* cysts (aQua Corporation, Osaka, Japan), as described previously (25). The bacterial 50 S ribosomal subunits and the 50 S core particles, which lack both L10 and L7/L12, were prepared form the L11-deficient *E. coli* strain AM68 (26), as previously described (27). The *B. mori–E. coli* hybrid 50 S particle was formed by mixing the *E. coli* 50 S core with the BmP0•BmP1•BmP2 stalk complex and BmL12 (5). The wild-type 50 S subunits were prepared from *E. coli* Q13 as described (27).

*Ribosome-inactivating toxin*. TCS was overexpressed in *E. coli* and purified as described previously (16).

### NMR analyses

*Structure determination of P1•P2 by NMR*. NMR spectra were collected in Bruker Avance 700 MHz spectrometers at 298 K. Protein samples of ^13^C,^15^N-HsP1**•**HsP2 and HsP1**•**^13^C,^15^N-HsP2 were used to obtain resonance assignment of HsP1 and HsP2, respectively. Sequential assignment of backbone resonances was obtained by Cα and Cβ connectivities generated by HNCACB and CBCA(CO)NH experiments. Side-chain resonances were obtained from TOCSY-HSQC, H(CC)CONH, HCCH-TOCSY and HCCH-COSY experiments. Inter-proton distance restraints were obtained from NOESY-type experiments such as ^1^H,^15^N-NOESY-HSQC, ^1^H,^13^C-NOESY-HSQC and ^1^H,^13^C-HSQC-NOESY-HSQC. Intermolecular distance restraints were obtained from the ^13^C-filtered/^13^C-edited NOESY experiment (28). Chemical shifts were referenced with respect to 4,4-dimethyl-4-silapentane-1-sulfonate. All multidimensional NMR data were processed with NMRPipe (29) and analyzed using NMRView (30). Dihedral angle restraints were derived from TALOS program (31). Hydrogen bond restraints were only included for the secondary structure elements. Structural calculation was performed using ARIA 2.2 (32) and CNS 1.2 (33,34) with an initial set of manually assigned nuclear Overhauser effects (NOEs). The structures were converged in the first round of calculation. ARIA-assigned NOEs were checked manually, and were included in subsequent rounds of calculation iteratively. Finally, 20 structures with the lowest total energy and no violation of experimental restraints (NOE or dihedral angle) were selected. Structural abnormalities in all stages were checked using PROCHECK (35).

*^15^N R1, R2 and heteronuclear NOE experiement*. Asymmetrically labeled ^15^N HsP1•HsP2 heterodimer was used to determine the ^15^N longitudinal relaxation rates (R_1_), transverse relaxation rates (R_2_) and heteronuclear NOE of HsP1•HsP2 heterodimer using Bruker Avance 700 MHz spectrometers at 298 K. Relaxation delays for R_1_ experiments were 0.011, 0.07, 0.128, 0.267, 0.533, 0.8 1.12, 1.44, 1.867 and 2.5 s, and for R_2_ experiments, the delays were 0.0001, 0.0005, 0.001, 0.0025, 0.005, 0.0075, 0.01, 0.015, 0.02, 0.03, 0.04, 0.05, 0.06, 0.08, 0.1, 0.12, 0.14 and 0.16 s. To evaluate ^1^H-^15^N NOEs, 2D spectra were recorded with and without NOE enhancement. Peak intensities in R_1_ and R_2_ experiments were fitted to mono-exponential equations in KaleidaGraph 4.0 (Abelbeck Software). Error for R_1_ and R_2_ values were estimated from the fitting routine in KaleidaGraph, which use the Levenberg–Marquardt algorithm. Errors in the ^1^H-^15^N NOE values were estimated from the root mean square noise of the spectra.

*Reduced spectral density mapping*. The spectral density at frequency zero, ω_N_ and ω_H_+ω_N_ are calculated by the following equations using the value of R_1_, R_2_ and heteronuclear NOE (36–38).

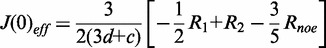


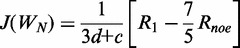


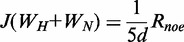

where 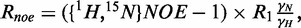 *d* = γ_H_^2^γ_N_^2^(h/2π)^2^/4r_HN_^6^, *c* = Δ^2^ω_N_^2^/3; Δ is the chemical shift anisotropy of the amide nitrogen; γ_H_ and γ_N_ are gyromagnetic ratio for nuclei ^1^H and ^15^N, respectively; h is the Planck’s constant; r_HN_ is the NH bond length. J(0)_eff_ is used instead of J(0), as contributions from other processes, such as chemical exchange R_ex_ (39) that increase the value of R_2_, are not explicitly considered in the present calculation. The effective correlation time of the backbone amide NH vectors were calculated from the following equation using the value of J(0)_eff_ (38):

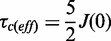



### Analysis for depurination at Sarcin/Ricin loop of 28 S/23 S rRNA

Ribosome samples (10 pmol each) were incubated with TCS in 50 µl of 5 mM MgCl_2_, 50 mM KCl, and 50 mM Tris–HCl (pH 7.6), at 37°C for 20 min, as indicated in legends for Figures 4 and 5. rRNA was extracted with phenol, and a portion of each sample was used as the template for primer extension, as described previously (40). Depurination at A2660 of 23 S rRNA or the corresponding base of 28 S rRNA was detected as a stop signal of primer extension by electrophoresis using either sequence gel (size 17 × 60 cm) or normal slab gel (size 14 × 13 cm), followed by autoradiography. Levels of depurination were estimated by intensity of radioactivity of the stop signals using the 23 S control primer for the 5′-end, as described previously (41).

### Model of P-complex recruiting TCS to the ribosome

HsP0 was modeled by MODELLER (42) using the crystal structure of archaeal *Pyrococcus horikoshii* aP0•aP1 complex (4) and *Methanococcus jannaschii* L10 (43) as templates. Helices 1–3 of HsP1•HsP2 heterodimer were docked to HsP0 model by superimposition to helices 1–3 of aP1 homodimer of archaeal aP0•aP1 complex. Helices 4 of human HsP1•HsP2 heterodimer were modeled according to helices 4 of archaeal aP1 (4). Models of the human P-complex were superimposed to the N-terminal domain of P0 in the crystal structure of yeast 80 S ribosome (44). Helices 4 of HsP1•HsP2 solution structure, together with the C-terminal tails, were aligned to helices 4 of the modelled HsP1•HsP2. Crystal structure of TCS complexed with C-terminal last 11 residues of HsP2 was aligned to the aspartate residues (Asp-106, Asp-107 and Asp-108 of HsP2) in the C-terminal tail of HsP1•HsP2 heterodimer.

## RESULTS

### P1•P2 heterodimer has a helical N-terminal domain and disordered C-terminal tails

**Figure 1.**
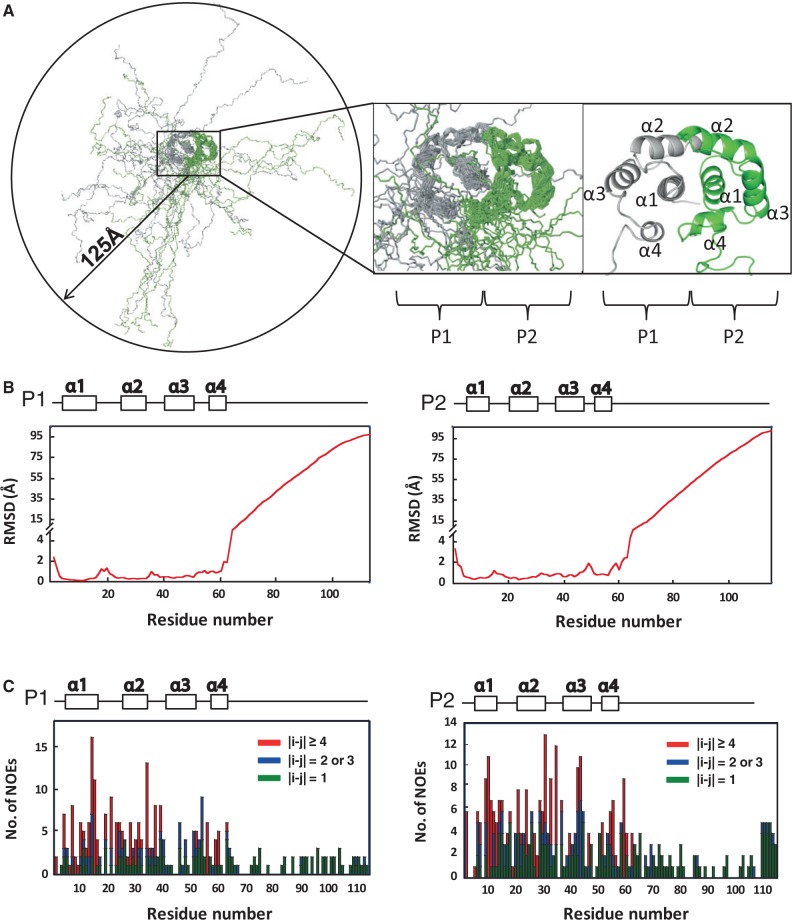
Solution structure of P1•P2. (**A**) Backbone overlay for the final ensemble of 20 structures of HsP1•HsP2 with the lowest energy and no restraint violation. The N-terminal dimerization domain (residue 1–62) is enlarged for clarity. The C-terminal tails are flexible and can extend to all directions with a radius of ∼125 Å (measured from the end of helix 4 to the C-terminal end of the farthest tail using PyMOL). (**B**) Backbone RMSD along the primary sequence. The N-terminal domains of the best 20 structures of HsP1•HsP2 were aligned, and the backbone RMSD values were calculated by MOLMOL. The N-terminal domain is well defined with an average backbone RMSD value of 0.67. In contrast, the C-terminal tails of HsP1•HsP2 are disordered, with RMSD values reaching ∼100 Å. (**C**) Number of sequential (green), short-range (blue) and long-range (red) NOEs along the primary sequence. The lack of long-ranged NOEs in the C-terminal tails of HsP1•HsP2 reflects that the region is disordered.

The N-terminal domain of HsP1•HsP2 heterodimer is responsible for the dimerization, and structure of this dimerization domain gives us insight on the assembly of the eukaryotic stalk (8). However, the C-terminal regions of P-proteins contain a highly conserved motif, which have been shown to bind translation factors (45) and ribosome-inactivating proteins (16,17). To better understand the structure-function of P1•P2, we have determined the structure of full-length HsP1•HsP2 heterodimer by NMR. The backbone overlay for the final ensemble of 20 structures with the lowest energy and no restraint violation are shown in Figure 1A, and statistics of structural calculation is summarized in Table 1. The structure of HsP1•HsP2 heterodimer can mainly be divided into two domains–a N-terminal dimerization domain (residue 1–62 of HsP1 and HsP2) and a flexible C-terminal tail, which is composed of the hinge and the highly conserved C-terminal regions (residue 63–114 of HsP1 and 63–115 of HsP2) (Figure 1A). The N-terminal domain of HsP1•HsP2 is well ordered, with an average backbone RMSD value of <1 Å (Figure 1B). The structure of the N-terminal domain is similar to that of the HsP1_ΔC_•HsP2_ΔC_ determined previously (8). In brief, both HsP1 and HsP2 have four helices in which their helices 1, 2 and 4 are facing each other at the dimeric interface. Helices 3 of both HsP1 and HsP2 are packed away from the interface and are not involved in dimerization (Figure 1A). On the other hand, no long-ranged NOEs were observed for the C-terminal tails of HsP1 and HsP2 (Figure 1C), resulting in disordered structure with backbone RMSD values reaching ∼100 Å at the C-termini (Figure 1B). As a result, the flexible tails of P1•P2 can extend up to ∼125 Å away from the N-terminal dimerization domain (Figure 1A).

**Table 1.** NMR and refinement statistics for the 20 structures of HsP1•HsP2 heterodimer with lowest energy and no restraint violation

NMR distance and dihedral restraints	
Distance restraints	
Total NOE	1451
Total unambiguous NOE	1318
Intramolecular	1206
Intra-residue	697
Sequential (|i-j| = 1)	321
Medium-range (1 < |i-j| < 5)	106
Long-range (|i-j| > 4)	82
Intermolecular	112
Total ambiguous NOE	133
Hydrogen bonds	118
Total dihedral angle restraints	262
Structure statistics	
Violations	
Distance restraints^a^ (Å)	0.053 ± 0.002
Dihedral angle restraint^a^ (°)	0.32 ± 0.11
No. of dihedral angle violation >5°	0
No. of distance restraint violation >0.5 Å	0
Deviation from idealized geometry	
Bond lengths^a^ (Å)	0.0041 ± 0.0001
Bond angles^a^ (°)	0.56 ± 0.01
Impropers^a^ (°)	1.44 ± 0.10
Average pairwise r.m.s. deviation (Å)	
Heavy^b^	1.103
Backbone^b^	0.674

^a^Values of mean and standard deviation were reported.

^b^r.m.s.d of the secondary structure elements of HsP1and HsP2 were reported.

### 15N relaxation analyses showed that the C-terminal tails of P1•P2 heterodimer are flexible

**Figure 2.**
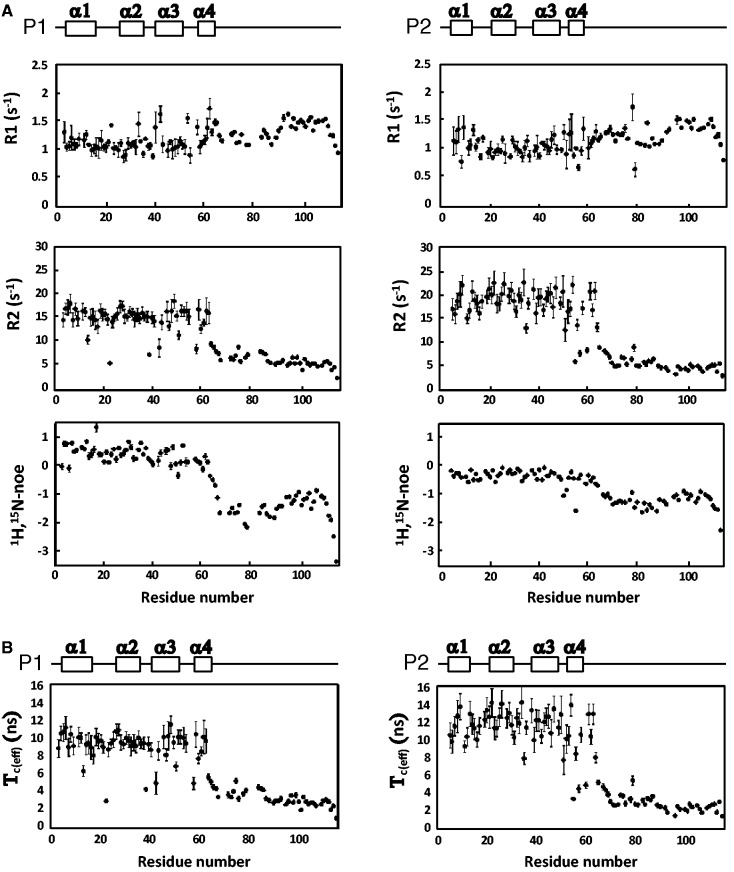
^15^N relaxation data suggest that the C-terminal tails of P1•P2 heterodimer are flexible. (**A**) ^15^N longitudinal (R_1_) and transverse (R_2_) relaxation rates, and f heteronuclear NOE for residues of HsP1 (left panel) and HsP2 (right panel) of the heterodimer. (**B**) Effective correlation time τ_c(eff)_, estimated by the spectral density mapping approach, for backbone amide of HsP1•HsP2.

To characterize the dynamics properties of HsP1•HsP2 heterodimer, we measured the ^15^N longitudinal (R_1_) and transverse (R_2_) relaxation rates and ^1^H-^15^N NOE (Figure 2A). It is apparent that residues from the C-terminal tail have relaxation parameters distinct from those of the N-terminal domain. For example, the C-terminal tail has a faster R_2_ of 6.1 ± 0.4 s^−^^1^ and a smaller ^1^H-^15^N NOE of −1.30 ± 0.01, compared with values of 17 ± 1 s^−^^1^ and 0.03 ± 0.06, respectively, for the N-terminal domain (Figure 2A). These observations indicate that the rate of rotational diffusion of N- and C-terminal halves should be different.

Here, we describe the dynamics of backbone amide of HsP1•HsP2 using an approach that map the spectral density function, J(ω), at three different frequencies: zero, ω_N_, ω_H_+ ω_N_ (corresponding to 0, 71, 629 MHz at a magnetic field of 17.6 Tesla) (Supplementary Figure S1A), and estimated the effective correlation time, τ_c(eff)_, for the reorientation of the backbone NH vector (Figure 2B). Residues in the C-terminal tails of HsP1•HsP2 heterodimer have significant lower values of J(0)_eff_ and higher values of J(629 MHz) (Supplementary Figure S1B). The resulting swallower spectral density map indicates that the C-terminal tails of HsP1•HsP2 are more mobile than the N-terminal domains of the dimer. This is evident from τ_c(eff)_ that the backbone amide groups in the N-terminal domains have a distinctly longer correlation time (10.1 ± 1.1 ns) than that in the C-terminal tails (3.34 ± 0.24 ns) (Figure 2B), suggesting that the residues in the C-terminal tails are flexible and re-orientate independently of the N-terminal dimerization domain.

### TCS can depurinate hybrid ribosomes carrying P1•P2 heterodimer but not *Escherichia coli* ribosomes

**Figure 3.**
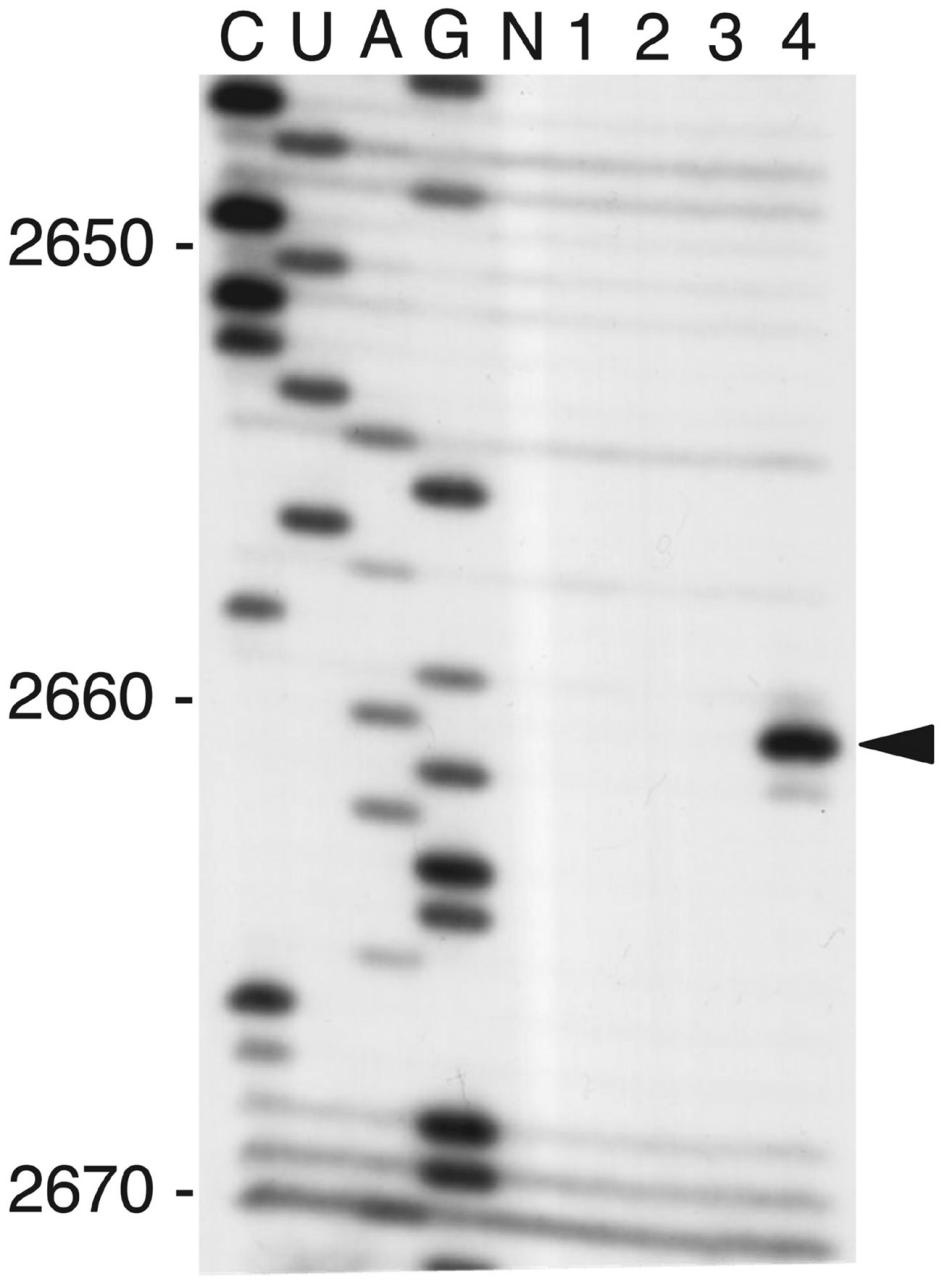
Eukaryotic P-complex, together with eL12, rendered *E. coli* ribosomes susceptible to depurination at A2660 by TCS. *E. coli* 50 S core (lane 1), which was deﬁcient in the *E. coli* L10•L7/L12 stalk complex and L11; *E. coli* intact 50 S (lane 2); the *in vitro*-reconstituted 50 S (lane 3), which was formed by mixing 50 S core (10 pmol); the *E. coli* L10•L7/L12 complex (20 pmol) and L11 (20 pmol); and the hybrid 50 S (lane 4), which was formed by mixing of 50 S core (10 pmol), the silkworm BmP0•BmP1•BmP2 complex (20 pmol) and BmL12 (20 pmol), were prepared individually. Each ribosome sample (10 pmol each) was incubated with 2.5 nmol of TCS. rRNAs were extracted and analysed by primer extension, followed by sequencing gel electrophoresis. Arrowhead indicates the position of A2660 in *E. coli* 23 S rRNA.

TCS is a RNA N-glycosidase that specifically inactivates eukaryotic ribosomes by depurination of a conserved adenine residue at the sarcin-ricin loop of the 28 S rRNA (46,47). We have previously shown that TCS binds to a consensus motif, SDDDMGFGLFD, located at the C-termini of P-proteins (16,17). Introduction of mutations that breaks the interaction between TCS and P-proteins impair its RNA N-glycosidase activity (16,17). Here, we hypothesize that the recognition of eukaryotic P1•P2 heterodimers as a complex with P0 provides the eukaryote-specific action of TCS to inactivate rRNA within the eukaryotic ribosome. To validate this hypothesis, we have attempted to reconstitute P0•P1•P2 stalk complex and to test its contribution in depurination of 23 S rRNA within *E. coli* 50 S core lacking L7/L12, L10 and L11. For this experiment, we used silkworm P-proteins BmP1, BmP2 and BmP0, as the conditions of reconstitution of the BmP0•BmP1•BmP2 complex and formation of an active hybrid ribosome, which is constructed with *E. coli* 50 S core and the BmP0•BmP1•BmP2 complex, supplemented with L11-like protein BmL12, have been well-established (Supplementary Figure S2). Primer extension analysis (Figure 3) showed that TCS was unable to depurinate *E. coli* 50 S ribosomes (lane 1) or 50 S core (lane 2) or *in vitro*-reconstituted 50 S (lane 3), which was formed by mixing 50 S core, the *E. coli* L10•L7/L12 complex and L11. In contrast, when the BmP0•BmP1•BmP2 complex and BmL12, which bind to the helices 42/43/44 of 28 S rRNA together (48), were added to 50 S core, TCS became to be able to depurinate A2660 base of 23 S rRNA in the hybrid 50 S ribosome (lane 4 of Figure 3, and also see Supplementary Figure S3). These observations suggest that replacement of the L10•L7/L12 stalk complex and L11 with the eukaryotic P-complex and eL12 rendered *E. coli* ribosome susceptible to the action of TCS.

### Depurination of the hybrid ribosome is mediated via the C-terminal tail of P-proteins

**Figure 4.**
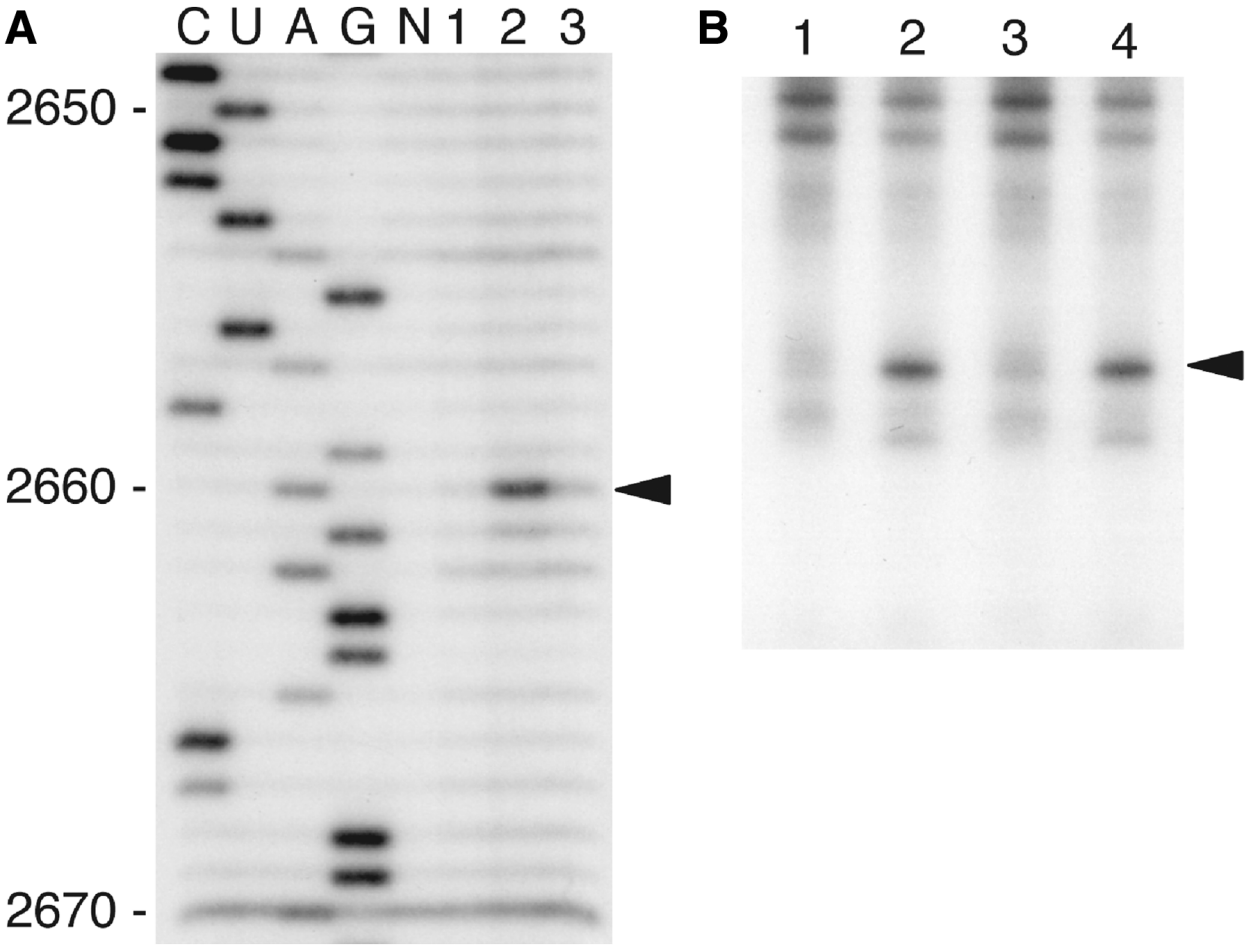
The C-terminal tails of the P-complex were essential for the depurination at A2660. (**A**) Effect of truncation of the C-terminal tails of the P-proteins on depuration at A2660 by TCS. *E. coli* 50 S core (lane 1); the hybrid 50 S carrying the intact P-proteins (lane 2), which was formed by mixing of 50 S core (10 pmol); the BmP0•BmP1•BmP2 complex (20 pmol) and BmL12 (20 pmol); and the hybrid 50 S carrying the truncated P-proteins (lane 3), which was formed by mixing of 50 S core (10 pmol), the BmP0_Δc_•BmP1_Δc_•BmP2_Δc_ complex (20 pmol) and BmL12 (20 pmol), were prepared individually. Each ribosome sample (10 pmol each) was incubated with 2.5 nmol of TCS. rRNAs were extracted and analysed by primer extension, followed by sequence gel electrophoresis. Arrowhead indicates the position of A2660 in *E. coli* 23 S rRNA. (**B**) Inhibition of TCS-dependent depurination of the eukaryotic 80 S ribosome by anti-P antibody that binds to the C-terminal conserved region of the P-complex. The *Artemia* 80 S ribosome samples (10 pmol each) were preincubated either without anitibody (lanes 1 and 2) or with 100 pmol of anti-P monoclonal immunoglobulin G (lane 3) and with 100 pmol of a control monoclonal immunoglobulin G, which does not react with any *Artemia* ribosomal proteins (lane 4). These ribosome samples were further incubated without (lane 1) or with 25 fmol of TCS (lanes 2–4). rRNA were extracted and analysed by primer extension, followed by slab-gel electrophoresis. Arrowhead indicates the position of base in 28 S rRNA, which corresponds to A2660 of *E. coli* 23 S rRNA.

To show that the C-terminal tails of P-proteins were essential for the depurination of rRNA by TCS, we reconstituted a P-complex with P-protein mutants [BmP1_ΔC_, BmP2_ΔC_ and BmP0_ΔC_ in which the C-terminal tails were truncated, as described previously (49)]. After TCS-treatment of the hybrid ribosomes carrying BmP0_ΔC_•BmP1_ΔC_•BmP2_ΔC_ or the wild-type BmP0•BmP1•BmP2 complex, the primer extension analysis was performed (Figure 4A, also see Supplementary Figure S3). The results showed that the depurination of A2660 by TCS, which was detected with the wild-type P-complex (Figure 4A, lane 2), was markedly reduced with the mutant P-complex lacking the C-terminal tails (Figure 4A, lane 3). To further support that the C-terminal tails are required for the action of TCS, we tested the depurination of *Artemia* ribosomes by TCS with or without anti-P monoclonal antibody (50) that binds to the conserved C-terminal region of P-proteins. As shown in Figure 4B, the depurination of intact 80 S ribosomes (lane 2) was inhibited by addition of anti-P (lane3), but not by the control monoclonal immunoglobulin G that is not reactive with any *Artemia* ribosomal proteins (lane 4). Taken together, our results suggest that the C-termainal tails of the P-complex were required for the recruitment of TCS to the sarcin-ricin loop to carry out its N-glycosidase activity.

**Figure 5.**
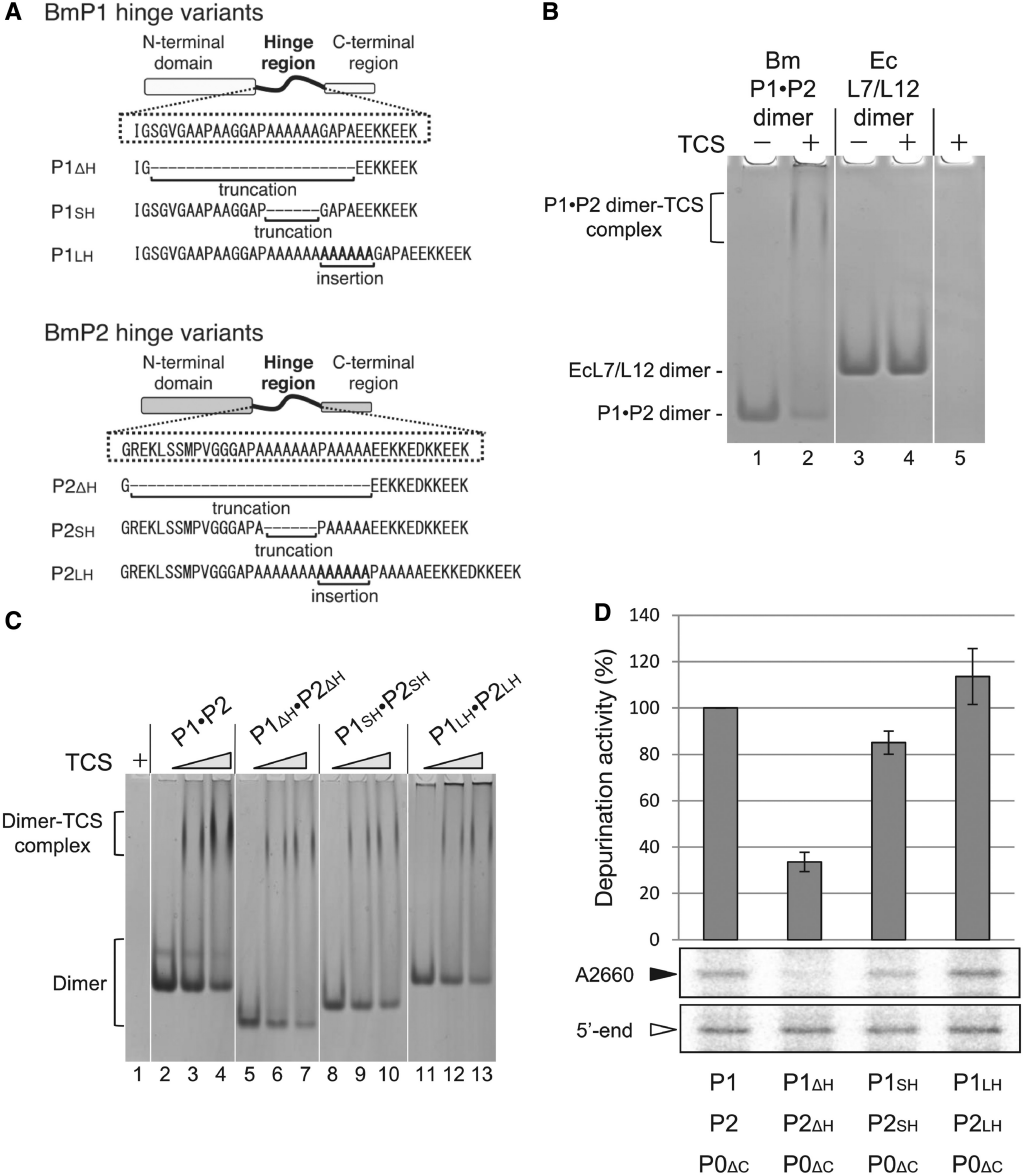
Depurination at A2660 of hybrid ribosome is dependent on the length of the hinge region of P-proteins. (**A**) Design of variants in the hinge regions of BmP1 and BmP2. (**B**) Silkworm BmP1•BmP2 heterodimer and *E. coli* (Ec) L7/L12 homodimer (200 pmol each) were incubated without (lanes 1 and 3) or with 1 nmol TCS (lanes 2 and 4) at 37°C for 10 min. The samples were subjected to 6% polyacrylamide gel electrophoresis at room temperature. Gel was stained with Coomassie Brilliant Blue. TCS (1 nmol) alone was also applied on lane 5. (**C**) 200 pmol each of wild-type BmP1•BmP2 heterodimer (lanes 2–4), BmP1_ΔH_•BmP2_ΔH_ (lanes 5–7), BmP1_SH_•BmP2_SH_ (lanes 8–10) and BmP1_LH_•BmP2_LH_ (lanes 11–13) was incubated without TCS (lanes 2, 5, 8 and 11), with 200 pmol of TCS (lanes 3, 6, 9 and 12) and with 600 pmol of TCS (lanes 4, 7, 10 and 13). TCS (600 pmol) alone (lane 1) was also incubated. All samples were separated by native gel electorophoresis as described in (B). (**D**) The stalk complexes were formed by incubation of BmP1•BmP2, BmP1_ΔH_•BmP2_ΔH_, BmP1_SH_•BmP2_SH_ or BmP1_LH_•BmP2_LH_ with P0_ΔC_ lacking C-terminal tail. The hybrid 50 S carrying each P-complex variant was formed by mixing 50 S core (10 pmol), each P-complex variant (20–60 pmol) and BmL12 (20 pmol). Each ribosome sample (10 pmol each) was incubated with 2.5 nmol of TCS. rRNAs were extracted and subjected to primer-extension analysis, followed by slab-gel electrophoresis (lower two panels). Two primers were used: one for the depurination at A2660 (black arrowhead) and the other for 5′-end of 23 S rRNA as a control (white arrowhead). Intensity of individual bands was measured by Bioimage Analyzer FLA-9000 (Fuji Photo Film). Individual values were normalized, and represented by bars (upper panel). The error bars indicate the variations of three independent assays.

The sarcin-ricin loop is located >80 Å away from the N-terminal domain of P1*•*P2. We hypothesize that the long hinge region of P1*•*P2 is essential for the recruitment of TCS, which binds to the consensus motif at the C-termini, to reach the sarcin-ricin loop. To test this hypothesis, we created variants of silkworm BmP1 and BmP2, in which the hinge regions were truncated (BmP1_ΔH_, BmP2_ΔH_), shortened (BmP1_SH_, BmP2_SH_) or lengthened (BmP1_LH_, BmP2_LH_) (Figure 5A). First, we checked binding of TCS to these stalk dimer variants by native gel electrophoresis (Figure 5B and 5C). As shown in Figure 5B, binding ability of TCS was detected with BmP1*•*BmP2 heterodimer (lane 2), but not with *E. coli* L7/L12 homodimer (lane 4). As shown in Figure 5C, TCS bound to all of BmP1_ΔH_*•*BmP2_ΔH_ (lanes 6, 7), BmP1_SH_*•*BmP2_SH_, (lanes 9, 10) and BmP1_LH_, BmP2_LH_ (lanes 12, 13) as well as wild-type BmP1*•*BmP2 (lanes 3, 4), suggesting that the hinge region of the stalk dimer is not involved in direct interaction with TCS. Next, we examine whether the hinge region contributes in the stalk-dependent depurination at A2660 using the hybrid ribosome. To focus on the roles of BmP1 and BmP2 variants, we used the anchor protein BmP0_ΔC_ lacking the C-terminal region. The complex formations of BmP0_ΔC_*•*BmP1_ΔH_*•*BmP2_ΔH_, BmP0_ΔC_*•*BmP1_SH_*•*BmP2_SH_ and BmP0_ΔC_*•*BmP1_LH_*•*BmP2_LH_ were confirmed without and with rRNA fragment covering P0/eL12-binding sites (Supplementary Figures S4A and S4B) by native-gel electrophoresis (48). It was also confirmed that they bound to 50 S core, together with BmL12 (Supplementary Figure S2), and approximately two copies of individual BmP1*•*BmP2 variants were assembled onto *E. coli* 50 S core by sucrose density gradient centrifugation (Supplementary Figure S5). Then, the depurination of these hybrid ribosome samples by TCS was analyzed by primer extension (Figure 5D). The results showed that, in contrast to binding data, truncation of the hinge region greatly reduced the depurination at A2660 of hybrid ribosome (see Figure 5D, BmP0_ΔC_*•*BmP1_ΔH_*•*BmP2_ΔH_). Moreover, the N-glycosidase activity of TCS at A2660 positively correlated with the length of the hinge region (Figure 5D, BmP0_ΔC_*•*BmP1_SH_*•*BmP2_SH_ and BmP0_ΔC_*•*BmP1_LH_*•*BmP2_LH_).

## DISCUSSION

Ribosome-inactivating proteins (RIPs) are N-glycosidase that inactivate ribosomes by depurinating a specific adenine residue (e.g. A2660 of 23 S rRNA in *E. coli* or A4324 of 28 S rRNA in rat) at the sarcin-ricin loop of rRNA (22,46,47,51,52). Such modification on rRNA renders the ribosome unable to bind elongation factors and halts protein synthesis (47,53). Although RIP can depurinate naked RNA, the N-glycosidase activity is 10^5^ time faster for rRNA within an intact ribosome (22), suggesting ribosomal proteins increase the susceptibility of rRNA toward RIPs. We have previously shown that TCS interacts with the C-terminal consensus motif (SDDDMGFGLFD) of eukaryotic stalk P-proteins (16,17). Breaking the interaction between TCS and P-proteins weakened the ribosome-inactivating and N-glycosidase activities (16,17). Besides, a number of other RIPs were found to interact with stalk proteins. For example, ricin-A-chain was found to interact with P0 by cross-linking in human lung carcinoma cell (54), and Shiga-like toxin 1 was found to interact with P0, P1 and P2 through *in vitro* pull-down assay (19). The C-terminal tail of stalk proteins was also shown to be the site of interaction with ricin-A-chain (19), maize RIP (20) and Shiga-like toxin 1 (19). The depurination activity of ricin-A-chain (18) and Shiga-like toxin 1 (55) on eukaryotic ribosome is greatly reduced in the absence of stalk proteins P1 and/or P2. In this report, we have shown that TCS required stalk proteins to depurinate the hybrid ribosome. These observations suggest the important role of stalk proteins in recruiting RIPs to the ribosome.

**Figure 6.**
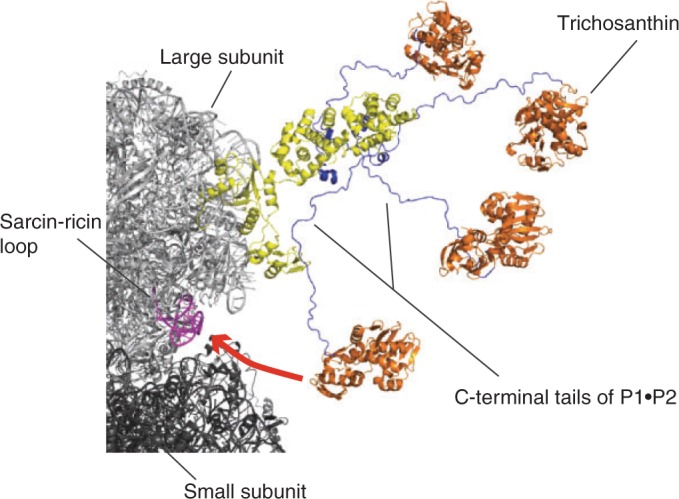
Structural model of P-complex recruiting TCS to the sarcin-ricin loop. The C-terminal tails (blue) of P-complex (yellow) point to different directions and extended from the N-terminal dimerization domain of the stalk. This allows the tails to cover a large area around the stalk for recruiting TCS (orange) to the sarcin-ricin loop (magenta).

In this study, we have determined the structure of full-length P1•P2 heterodimer by NMR spectroscopy, and showed that the N-terminal domain is structured and is responsible for dimerization. Supported by ^15^N relaxation study, we showed that the C-terminal tails of P1•P2 are flexible and can extend up to ∼125 Å away from the dimerization domain (Figure 1). In the recently determined crystal structure of yeast ribosome, only the N-terminal dimerization domain of one copy of P1•P2 dimer was observed (44). The intrinsic flexibility of the C-terminal tails of P-proteins observed in this study explains why it was difficult to define the crystal structure of P-proteins in the eukaryotic ribosome. We have previously determined the crystal structure of TCS in complex with the C-terminal conserved motif, SDDDMGFGLFD, of P-proteins and showed that these residues can adopt a defined structure upon complex formation (17). Together with the solution structure of full length P1•P2 reported in this study, we were able to build a structural model of how the eukaryotic stalk proteins can help to recruit TCS to the sarcin-ricin loop (Figure 6). Multiple copies of the C-terminal tails can cover a large space around the N-terminal dimerization domain of the stalk, which should increase the chance of catching TCS. This is in agreement with a previous study that the rate of association of ricin-A-chain with the pentameric P0(P1•P2)_2_ was higher than that of trimeric P0(P1•P2) (56). As the C-terminal tails are long and flexible, they can extend far away from the N-terminal dimerization domain of P1•P2 and present the consensus motif at the C-termini to all directions to facilitate TCS binding. After TCS binding, the C-terminal tails are long enough to recruit TCS to the sarcin-ricin loop, where the RIP carries out its N-glycosidase activity (Figure 6). This model is supported by the observation that shortening of the C-terminal tails by truncating the hinge region greatly reduced the depurination at A2660 of the hybrid ribosome (Figure 5).

Moreover, we also demonstrate that the interaction of TCS with eukaryotic stalk P-proteins is involved in the eukaryote-specific action of TCS. Replacing the bacterial L10(L7/L12)_4_/L11 proteins with eukaryotic P0(P1•P2)_2_/eL12 proteins rendered the *E. coli* ribosome susceptible to the N-glycosidase activity of TCS (Figure 3), but not with the P-complex composed of the truncation mutants of P0, P1 and P2, in which the C-terminal tails were truncated. Eukaryotic and bacterial stalk proteins are structurally distinct. The solution structure of full-length P1•P2 reported here reveals that the most striking difference between L7/L12 homodimer and P1•P2 heterodimer is at their C-terminal parts (Supplementary Figure S6). The structure of the C-terminal region of L7/L12 is a globular domain consisted of 3 α-helices and 3 β-strands (57,58). ^15^N relaxation study of L7/L12 showed that the R_1_, R_2_ and heteronuclear NOE values are consistent with a compact well-ordered globular structure (57,58). In contrast, in this study, we showed that the C-terminal parts of P1 and P2 are flexible and unfolded. The role of the C-terminal tails of P1•P2 in facilitating the depurination action of TCS is supported by the fact that truncation of the C-terminal tails in the P-complex resulted in a hybrid ribosome that is not susceptible to TCS (Figure 4 and Supplementary Figure S3). The structural difference between eukaryotic and bacterial stalk proteins justified the eukaryote-specific recognition of TCS toward eukaryotic ribosome. As shown in Figure 5B, TCS can only interact with eukaryotic stalk P1•P2 proteins, but not with bacterial stalk proteins. Taken together, our results are consistent with the conclusion that the eukaryotic stalk proteins play an essential role in rendering ribosome susceptible to eukaryote-specific ribosome-inactivating proteins like TCS and ricin-A-chain.

Interestingly, the modes of recruitment of TCS and elongation factors to ribosomes share many similarities. Both involve binding to the sarcin-ricin loop and to the stalk proteins. It has been shown previously that ribosome stalk provide the domain-specific binding and utilization of elongation factors among the three domains of life (3,27,45,59). For example, it has been shown that the C-terminal domains of L7/L12 binds bacterial elongation factors (3), and the C-terminal conserved region of archaeal P1 bind archaeal aEF-1α, aEF-2 and aIF5B (45). Consistent with these observations, we showed that the C-terminal tail of P1 or P2 plays a similar role in binding eukaryotic eEF-2 (Supplementary Figure S7). In an analogy, in this study, we showed that the C-terminal tails of eukaryotic stalk proteins play an essential role in eukaryote-specific binding of TCS to ribosome. It is likely that the recruitment of elongation factors to the ribosome adopts a mechanism similar to the way that TCS recruited by eukaryotic stalk proteins. Moreover, eukaryote-specific ribosome-inactivating proteins, e.g. TCS and ricin, may have evolved for exploiting the stalk-dependent translation-factor-recruiting machinery of the eukaryotic ribosome to exert their specific and efficient action.

Apart from interacting with stalk proteins, some RIPs may facilitate their inactivation via other ribosomal proteins. For example, the toxicity and depurination activity of Shiga-like toxin 2 toward ribosome with P0 lacking P1•P2 binding sites were found similar to that of the wild-type ribosomes, suggesting the toxin can still exert its function with an incomplete stalk (55). Moreover, a monoclonal antibody that binds to the C-terminal tail of the P-proteins could protect ribosome from inactivation by TCS but not PAP, showing that PAP does not need the C-terminal tail to function (60). Interestingly, PAP can inactivate eukaryotic as well as prokaryotic ribosomes. It has been reported that PAP can interact with ribosome L3 (21). The ability of PAP to depurinate both prokaryotic and eukaryotic ribosomes can be justified by its interaction with L3, which is highly conserved in eukaryotic and prokaryotic ribosomes (18). Apparently, the dual-specific PAP has evolved other mechanisms to facilitate their actions. Moreover, we noticed that much lower amount of TCS was sufficient to depurinate *Artemia* ribosome than that required for the same amount of the hybrid ribosome, which contains a core body from *E. coli* 50 S subunits and a eukaryotic P0(P1•P2)_2_/eL12 stalk complex. This observation suggests that interaction of TCS/stalk-complex with other ribosomal elements around the sarcin/ricin loop may further facilitate the action of TCS. Identification of such elements is an interesting point to be addressed in future.

## ACCESSION NUMBERS

Atomic coordinates and NMR restraints for the reﬁned structures have been deposited to Protein Databank in Europe (PDBe) with wwPDB ID code 4beh and r4behmr, respectively.

## SUPPLEMENTARY DATA

Supplementary Data are available at NAR Online, including [61].

## FUNDING

General Research Fund [Project no. 477509 to K.-B. W.] from the Research Grants Council of Hong Kong SAR; Special Equipment Grants [Project no. SEG/CUHK09 to K.-B. W.] from the University Grants Committee of Hong Kong SAR; a Grant-in-Aid for Scientific Research (B) [24370073 to T. U.] from the Ministry of Education, Culture, Sports, Science, and Technology of Japan; a postdoctoral fellowship from the Chinese University of Hong Kong (to K.-M.L.). Funding for open access charge: The Chinese University of Hong Kong.

*Conflict of interest statement*. None declared.

## Supplementary Material

Supplementary Data
