# Supplementary material for: Solution structure of human P1•P2 heterodimer provides insights into the role of eukaryotic stalk in recruiting the ribosome-inactivating protein trichosanthin to the ribosome
Source: Nucleic Acids Res. 2013 Jul 26;41(18):8776–87. doi: 10.1093/nar/gkt636 (PMC3794596; doi:10.1093/nar/gkt636)
Supplement: Supplementary Data [file supp_41_18_8776__index.html]

Solution structure of human P1•P2 heterodimer provides insights into the role of eukaryotic stalk in recruiting the ribosome-inactivating protein trichosanthin to the ribosome — Solution structure of human P1•P2 heterodimer provides insights into the role of eukaryotic stalk in recruiting the ribosome-inactivating protein trichosanthin to the ribosome — Solution structure of human P1•P2 heterodimer provides insights into the role of eukaryotic stalk in recruiting the ribosome-inactivating protein trichosanthin to the ribosome — Supplementary Data 

# Solution structure of human P1•P2 heterodimer provides insights into the role of eukaryotic stalk in recruiting the ribosome-inactivating protein trichosanthin to the ribosome

## 

files

**Files in this Data Supplement:**

- Supplementary Data - pdf file
