# Supplementary material for: Solution structure of human P1•P2 heterodimer provides insights into the role of eukaryotic stalk in recruiting the ribosome-inactivating protein trichosanthin to the ribosome
Source: Nucleic Acids Res. 2013 Jul 26;41(18):8776–87. doi: 10.1093/nar/gkt636 (PMC3794596; doi:10.1093/nar/gkt636)
Supplement: Supplementary Data [file supp_gkt636_nar-00899-r-2013-File009.pdf]

SUPPLEMENTARY DATA

Solution structure of human P1•P2 heterodimer provides insights into the role of eukaryotic stalk in recruiting ribosome-inactivating protein trichosanthin to the ribosome

Ka-Ming Lee[1,3], Kazuyuki Yusa[2,3], Lai-On Chu[1], Conny Wing-Heng Yu[1], Moe Oono[2], Tomohiro Miyoshi[2], Kosuke Ito[2], Pang-Chui Shaw[1], Kam-Bo Wong[1,*] and Toshio Uchiumi[2,*]

[1] School of Life Sciences, Centre for Protein Science and Crystallography, The Chinese University of Hong Kong, Shatin, Hong Kong, China

[2] Department of Biology, Faculty of Science, Niigata University, Ikarashi 2-8050, Nishi-ku, Niigata 950-2181, Japan.

[3] Ka-Ming Lee and Kazuyuki Yusa are joint First-Authors of this work.

* To whom correspondence should be addressed:

Kam-Bo Wong, Email: kbwong@cuhk.edu.hk

Room 289, Science Centre, School of Life Sciences, The Chinese University of Hong Kong, Shatin, Hong Kong, China

Toshio Uchiumi, Email: uchiumi@bio.sc.niigata-u.ac.jp

Department of Biology, Faculty of Science, Niigata University, Ikarashi 2-8050, Nishi-ku, Niigata 950-2181, Japan.

SUPPLEMENTARY METHODS

Sample preparation

Elongation factor 2 (eEF-2). Livers from adult Sprague-Dawley rats were homogenized with 0.5 mM PMSF in homogenizing buffer (0.27 M sucrose, 30 mM KCl, 5 mM magnesium acetate, 1 mM DTT, 20 mM Tris-HCl, pH 7.6) (2.0-2.5 ml/g of tissue) using an electronic tissue tearer. The lysate was centrifuged at 27,000 g for 30 minutes to remove cell debris. The supernatant was then precipitated using ammonium sulfate. Precipitate that formed above 40 % saturation but below 60 % saturation was collected. The pellet collected was resuspended with minimal volume of buffer A (10 % glycerol, 50 mM KCl, 0.1 mM EDTA, 14 mM β-mercaptoethanol, 20 mM Tris-HCl, pH 7.6) and dialyzed against buffer A at 4 ºC overnight. The dialyzed sample was centrifuged at 27,000 g for 30 minutes to remove unwanted protein precipitate. The supernatant was filtered through 0.22 μm filter (Millipore) and loaded to 100 ml DEAE Sepharose fast flow resin (GE Healthcare) equilibrated with Buffer A packed in Biorad Econo column (2.5 cm x 30 cm). After extensive washing, proteins bound to DEAE column were step-eluted with Buffer A with 150 mM KCl. The fraction collected from DEAE chromatography was then directly loaded to 5 ml Hi-Trap Heparin column (GE Healthcare) equilibrated with Buffer A. After extensive washing, a 200 ml linear 50-500

mM KCl gradient was applied. Fractions containing eEF-2 were concentrated to 5 ml and loaded to HiLoad superdex 200 gel filtration column equilibrated in Buffer A. The fractions containing eEF-2 was loaded to Mono Q HR 16/10 column (GE Healthcare) and purified eEF-2 was eluted by 200 ml linear gradient of 50-500 mM KCl. All the homogenizing and centrifugation procedures were done at 0 – 4ºC.

In-vitro pull-down assay

HsP1•HsP2 and $HsP1_{\Delta C}$•$HsP2_{\Delta C}$ heterodimer or C-terminal 36 amino acids of HsP2 fused with MBP (MBP-C36) were applied and coupled to NHS-activated sepharose (GE Healthcare) according to manufacturer instruction. About 10 μg of eEF-2 was loaded to 100 μl of protein-coupled NHS-activated sepharose equilibrated with binding buffer (10 mM NaCl, 20 mM Tris-HCl, pH 8.0). The resin was incubated at room temperature for 30 minutes for binding. After extensive washing, proteins were eluted by elution buffer (1M NaCl, 20 mM Tris-HCl, pH 8.0). The presence of eEF-2 in the elution was detected by western blotting using anti-eEF-2 antibodies.

SUPPLEMENTARY RESULTS

The C-terminal tail is responsible for binding elongation factor 2

It has been previously shown that the C-terminal tails of archaeal P-protein can bind

elongation factors (1). Given the sequence homology between archaeal and eukaryotic P-proteins, it is likely that the C-terminal tails of eukaryotic P-proteins are also responsible for binding elongation factors. To test this hypothesis, full-length HsP1·HsP2 heterodimer and its dimerization domain $HsP1_{\Delta C}$·$HsP2_{\Delta C}$ heterodimer were coupled to NHS-activated resin and the interaction with rat elongation factor 2 (eEF-2) were tested using *in vitro* pull down assay. The eluted fractions were analyzed by western blot using anti-eEF-2 antibody (Supplementary Figure S7A). eEF-2 was found in a fraction eluted form full-length HsP1·HsP2 heterodimer but not from $HsP1_{\Delta C}$·$HsP2_{\Delta C}$, showing that the C-terminal tail is important in binding eEF-2. Next, we tested the interaction between eEF-2 and the C-terminal 36 residues of P2 fused with maltose-binding-protein (MBP-C36) by in-vitro pull down assay (Supplementary Figure S7B). We found that MBP-C36, but not MBP, interacts with eEF-2 (Supplementary Figure S7B). Consistent with the observation for archaeal P-proteins (1), the C-terminal tails of eukaryotic P-proteins are responsible for direct interaction with eEF-2.

SUPPLEMENTARY TABLE

Primer name	Sequence（5’ → 3’）
BmP1$_{\Delta H}$ F	GAGGAGAAGAAGGAAGAAAA
BmP1$_{\Delta H}$ R	GCCGATGTTGGTGATCAGGT
BmP1$_{SH}$ F	GGCGCTCCGGCCGAGGAGAAGAAG
BmP1$_{SH}$ R	TGGCGCTCCACCAGCGGCCGGAGCAGCA
BmP1$_{LH}$ F	GCAGCAGCAGCTGCAGCCGGCGCTCCGGCCGAGG
BmP1$_{LH}$ R	TGCTGCTGCAGCAGCGGCTGGCGCTCCACCAGCG
BmP2$_{\Delta H}$ F	GAGGAGAAGAAAGAGGACAAGAAAGAGGAGAA
BmP2$_{\Delta H}$ R	TCCGGCCGCAATAAGTTGTTCAAC
BmP2$_{SH}$ F	CCAGCTGCTGCTGCTGCTGAGGAGAAGA
BmP2$_{SH}$ R	TGCAGGTGCTCCGCCTCCTA
BmP2$_{LH}$ F	GCAGCAGCAGCAGCTGCCCCAGCTGCTGCTGCTG
BmP2$_{LH}$ R	TGCTGCTGCAGCAGCCGCTGCAGGTGCTCCGCCT

Supplementary Table S1. Oligonucleotide primers

F indicates forward primer, R indicates reverse primer. The inserted nucleotides are underlined.

SUPPLEMENTARY FIGURES

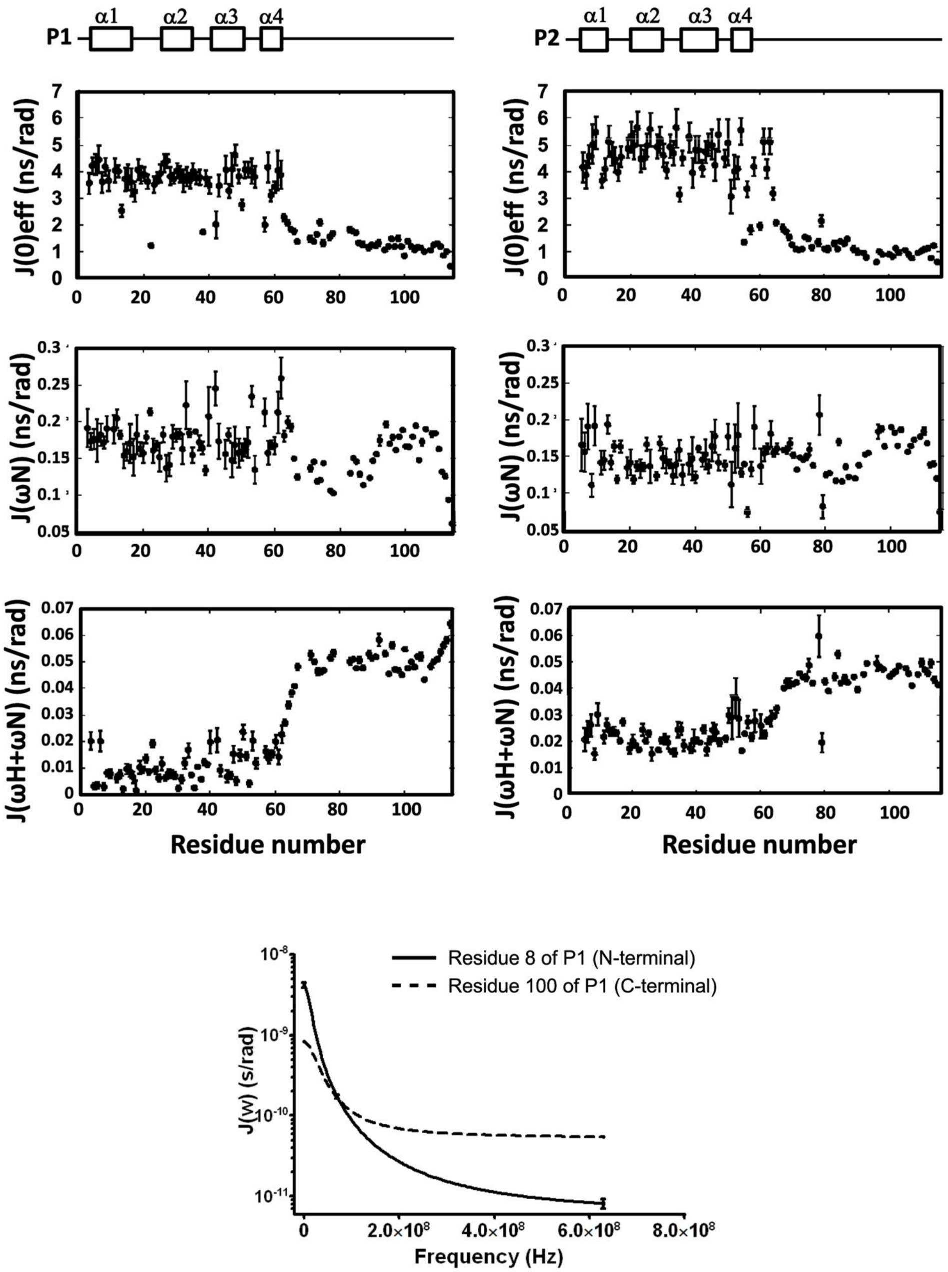

Supplementary Figure S1. Spectral density approach for studying dynamics of HsP1•HsP2.

(A) Spectral density function at J(0) eff, J(71MHz), J(629MHz) of HsP1•HsP2 heterodimer estimated by the reduced spectral density mapping approach (2-4). (B) Spectral density map of typical residues at N- and C-terminal domains of HsP1•HsP2. It is noted that residue 100 in the C-terminal tail of HsP1 has a swallower spectral density function than that of residue 8 in the N-terminal domain of HsP1.

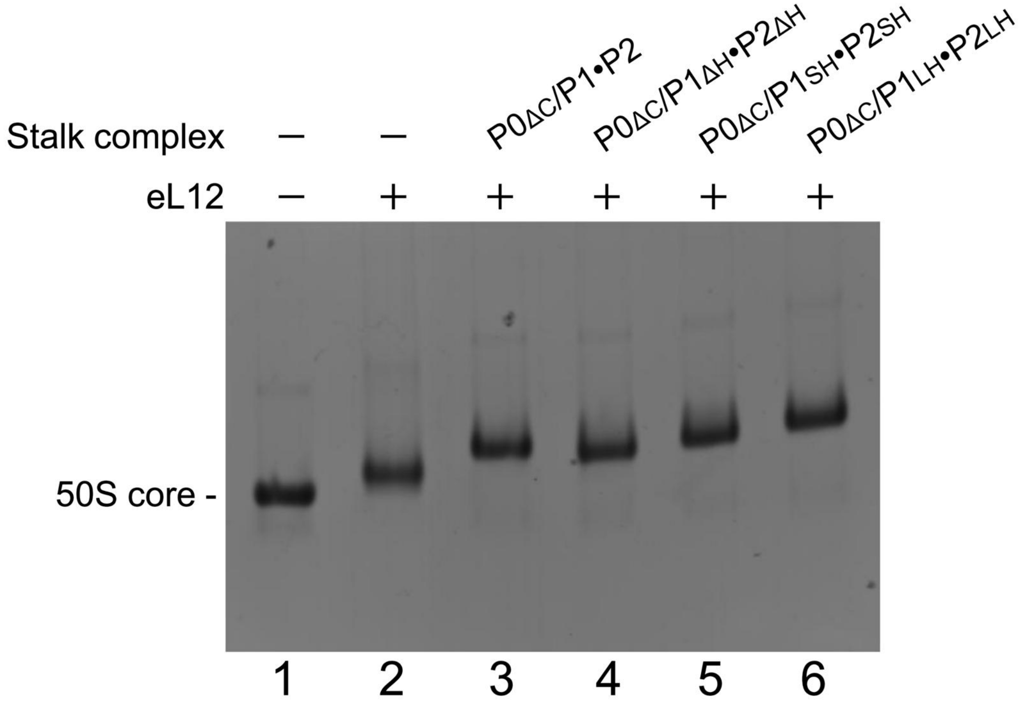

Supplementary Figure S2. Formations of hybrid 50S particles composed of *E. coli* 50S core and silkworm stalk complex variants.

The *E. coli* 50S core (5 pmol, lane 1) deficient in L10•L7/L12 stalk complex and L11was incubated with 10 pmol BmL12 (lane 2), or the same amount of BmL12, together with 10 pmol each of the BmP0$_{\Delta C}$•BmP1•BmP2 (lane 3), BmP1$_{\Delta H}$•BmP2$_{\Delta H}$ (lane 4), BmP1$_{SH}$•BmP2$_{SH}$ (lane 5) and BmP1$_{LH}$•BmP2$_{LH}$ (lane 6). These samples were analyzed by acrylamide-agarose composite gel electrophoresis, as described previously (5,6). The gel was stained with Azur B.

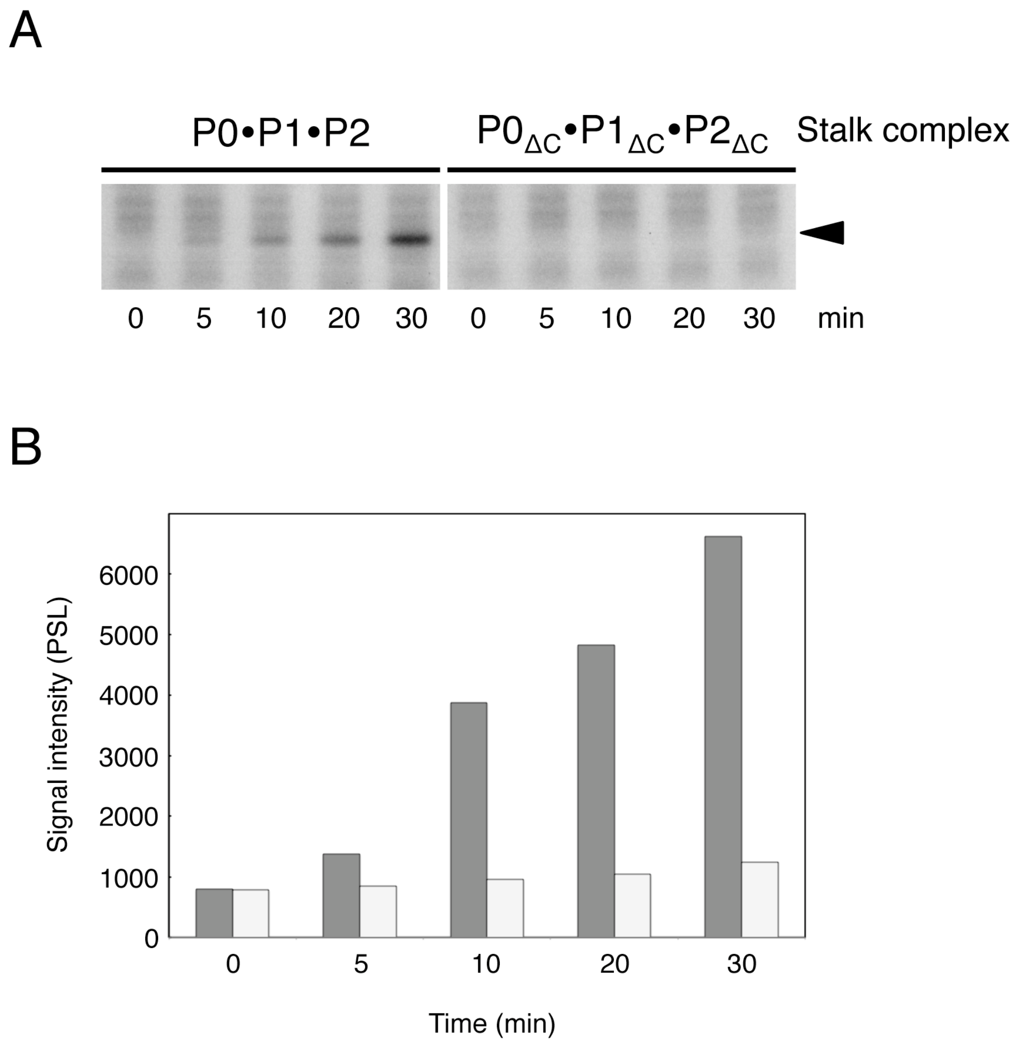

Supplementary Figure S3. Time course of depurination of A2660 in the hybrid ribosome by trichosantin.

(A) The hybrid 50S was formed by mixing of 50S core (10 pmol), BmL12 (20 pmol), and 20 pmol of either the intact BmP0•BmP1•BmP2 stalk complex (left) or the truncated $BmP0_{\Delta c}$•$BmP1_{\Delta c}$•$BmP2_{\Delta c}$ stalk complex (right). Each ribosome sample (10 pmol each) was incubated with 2.5 nmol of TCS for 0, 5, 10, 20, or 30 min. Individual rRNAs were extracted and analysed by primer extension, followed by slab-gel electrophoresis and autoradiography. Arrowhead indicates the position of A2660 in *E. coli* 23S rRNA. (B) Intensity of individual bands on the gels (A) was quantified by using Bioimage Analyzer and Multi Gauge software (Fuji Film), as levels of photo stimulated luminescence (PSL). Dark bars and white bars indicate levels of the depurination in hybrid 50S carrying the intact stalk complex and the truncated stalk complex, respectively.

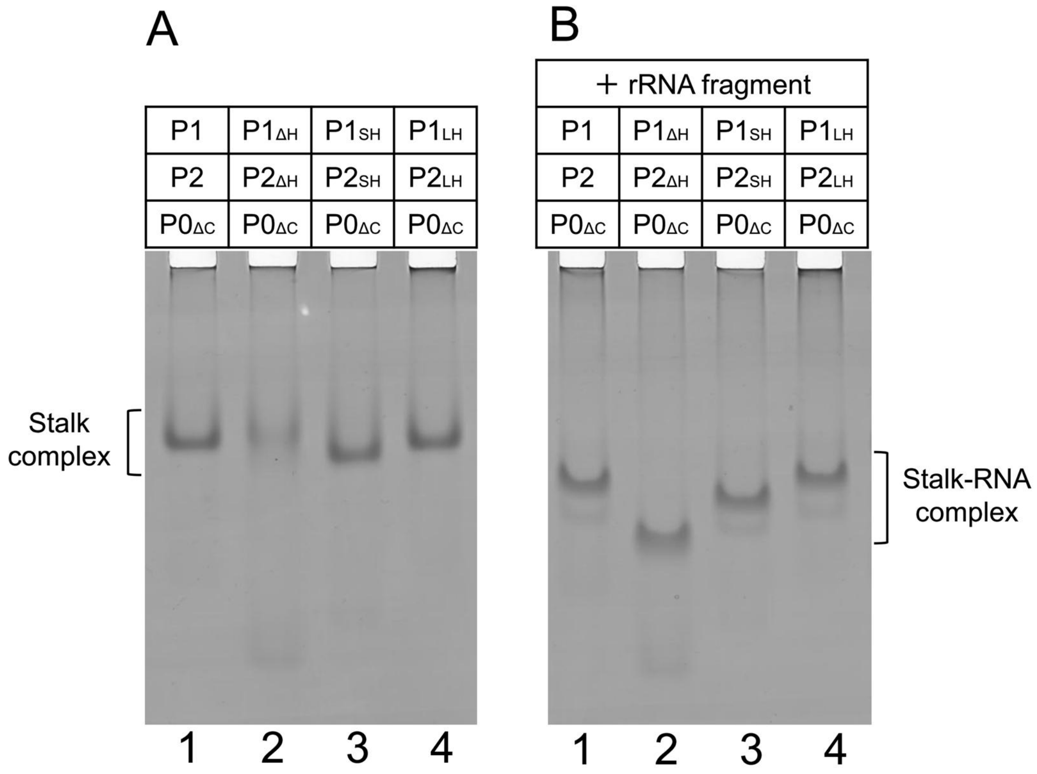

Supplementary Figure S4. Formations of silkworm stalk complexes with mutations at the hinge regions of P1 and P2.

(A) The complexes were reconstituted individually by incubation of 100 pmol $BmP0_{\Delta C}$ with 300 pmol of each variant of the stalk dimer: BmP1•BmP2 (lane 1), with $BmP1_{\Delta H}$•$BmP2_{\Delta H}$ (lane 2), with $BmP1_{SH}$•$BmP2_{SH}$ (lane 3), and with $BmP1_{LH}$•$BmP2_{LH}$ (lane 4). Individual complexes were subjected to the native gel electrophoresis, as described previously (6). The gel was stained with Coomassie Brilliant Blue. (B) The complexes were reconstituted under the same condition as (A), except that the complex formation was performed in the presence of 200 pmol of the rRNA fragment including H41/42/43 of rat 28S rRNA (7). Individual complexes were subjected to native gel electrophoresis. The gel stained with Coomassie Brilliant Blue.

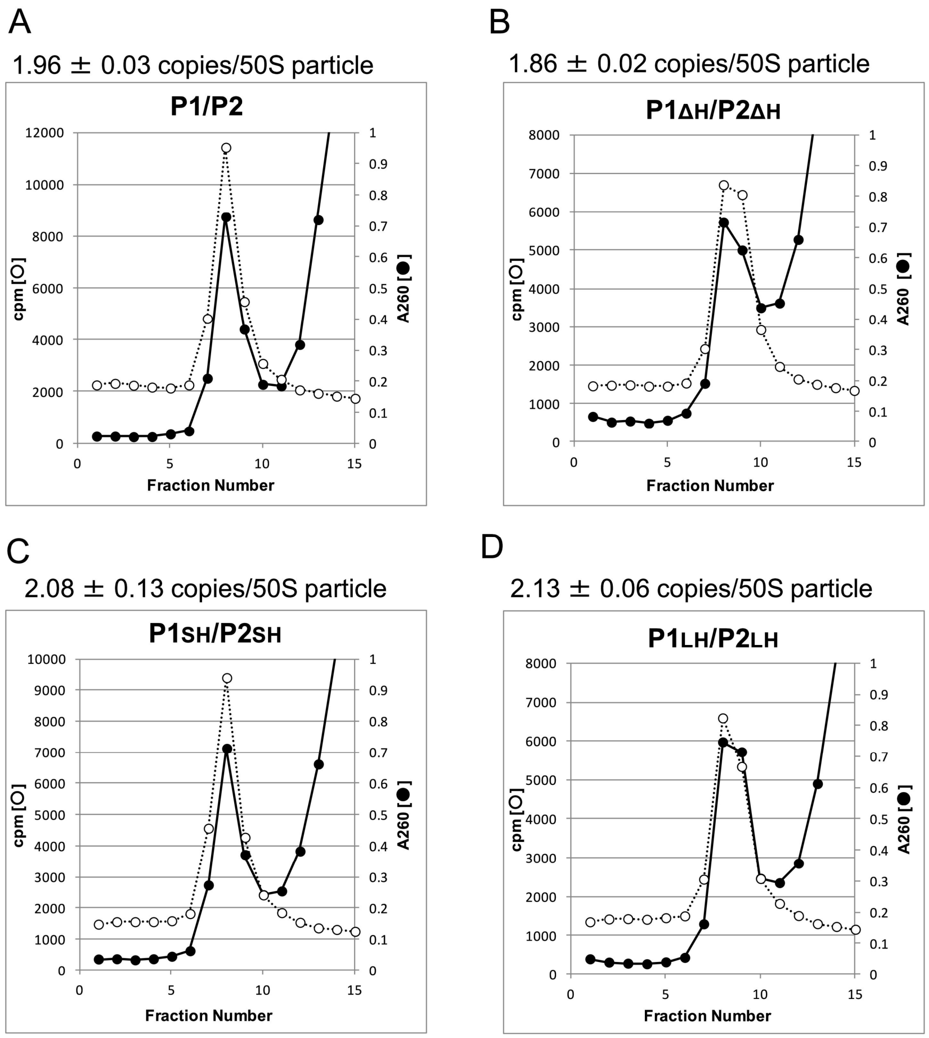

Supplementary Figure S5. Quantitative analysis of stalk dimers carrying mutations in hinge region bound to the ribosome.

(A) The 50S core (30 pmol) was incubated with excess amounts of the BmP0$_{\Delta C}$•BmP1•BmP2 complex (90 pmol), whose BmP1 component had been labeled with ^{32}P (279 - 418 cpm/pmol) (6), and BmL12 (90 pmol). The other hybrid 50S particles were also formed as (A), except that instead of BmP0$_{\Delta C}$•[^{32}P]BmP1•BmP2, BmP0$_{\Delta C}$•[^{32}P]BmP1$_{\Delta H}$•BmP2$_{\Delta H}$ (B), BmP0$_{\Delta C}$•[^{32}P]BmP1$_{SH}$•BmP2$_{SH}$ (C), and BmP0$_{\Delta C}$•[^{32}P]BmP1$_{LH}$•BmP2$_{LH}$ (D). The hybrid 50S samples were separated by sucrose gradient centrifugation, as described previously (6,8). Typical sedimentation patterns of individual hybrid sample were indicated. The amounts of BmP1•BmP2 dimer and its variants incorporated to the 50S core were estimated by radioactivity of [^{32}P]P1 and the absorbance at 260 nm of the 50S core. Copy numbers for BmP1 or its variants bound to the 50S core, which are means of three experiments, were shown in the upper part of individual panels.

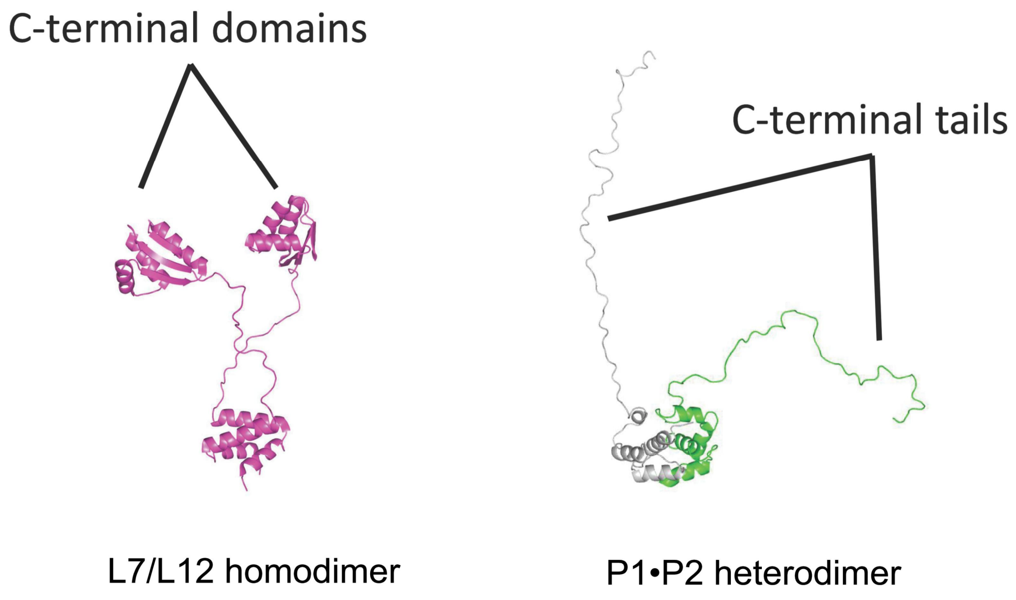

Supplementary Figure S6. Comparison of the solution structures of L7/L12 homodimer (9) and P1・P2 heterodimer.

L7 homodimer has a structured C-terminal domain while P1・P2 heterodimer has a flexible C-terminal tail.

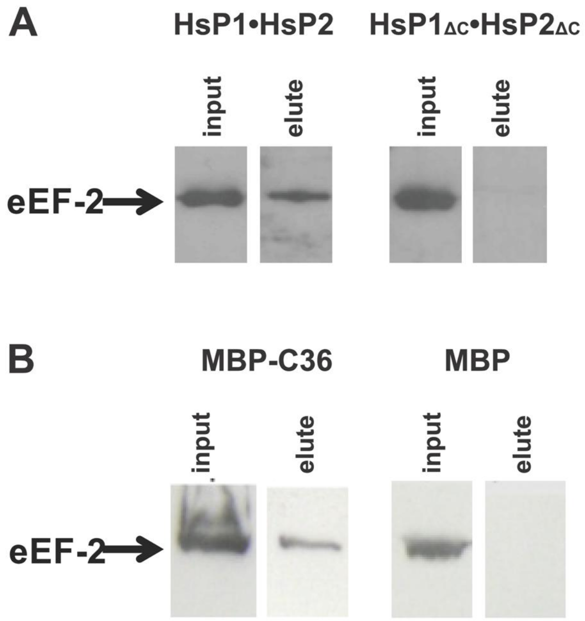

Supplementary Figure S7. eEF-2 interacts with C-terminal tail of P2. (A) HsP1•HsP2 and $HsP1_{\Delta C}$·$HsP2_{\Delta C}$ heterodimer were coupled to NHS-activated resin. eEF-2 was loaded to coupled resin. After extensive washing and elution, the result was analyzed by western blotting with 10% SDS-PAGE. The presence of eEF2 at the eluted fraction of HsP1•HsP2-coupled resin rather than $HsP1_{\Delta C}$·$HsP2_{\Delta C}$-coupled resin indicated that eEF-2 interacted with C-terminal tail. (B) To further confirm the interaction, instead of coupling P1•P2 or its dimerization domain to NHS-activated resin, C-terminal peptides of HsP2 were fused with MBP and coupled to the NHS-activated resin (MBP-C36 means the last 36 residues from the C-terminus of P2 were fused with MBP). MBP was coupled to the resin as a negative control. The presence of eEF-2 in the elution of MBP-C36 coupled resin showed that eEF-2 interacts directly with the C-terminal tail.

SUPPLEMENTARY REFERENCES

1. Nomura, N., Honda, T., Baba, K., Naganuma, T., Tanzawa, T., Arisaka, F., Noda, M., Uchiyama, S., Tanaka, I., Yao, M. *et al.* (2012) Archaeal ribosomal stalk protein interacts with translation factors in a nucleotide-independent manner via its conserved C terminus. *Proc Natl Acad Sci U S A*, **109**, 3748-3753.
2. Farrow, N.A., Zhang, O., Forman-Kay, J.D. and Kay, L.E. (1995) Comparison of the backbone dynamics of a folded and an unfolded SH3 domain existing in equilibrium in aqueous buffer. *Biochemistry*, **34**, 868-878.
3. Ishima, R. and Nagayama, K. (1995) Protein backbone dynamics revealed by quasi spectral density function analysis of amide N-15 nuclei. *Biochemistry*, **34**, 3162-3171.
4. Peng, J.W. and Wagner, G. (1995) Frequency spectrum of NH bonds in eglin c from spectral density mapping at multiple fields. *Biochemistry*, **34**, 16733-16752.
5. Tokimatsu, H., Strycharz, W.A. and Dahlberg, A.E. (1981) Gel electrophoretic studies on ribosomal proteins L7/L12 and the Escherichia coli 50 S subunit. *J Mol Biol*, **152**, 397-412.
6. Hagiya, A., Naganuma, T., Maki, Y., Ohta, J., Tohkairin, Y., Shimizu, T., Nomura, T., Hachimori, A. and Uchiumi, T. (2005) A mode of assembly of P0, P1, and P2 proteins at the GTPase-associated center in animal ribosome: in vitro analyses with P0 truncation mutants. *J. Biol. Chem.*, **280**, 39193-39199.
7. Uchiumi, T. and Kominami, R. (1997) Binding of mammalian ribosomal protein complex P0.P1.P2 and protein L12 to the GTPase-associated domain of 28 S ribosomal RNA and effect on the accessibility to anti-28 S RNA autoantibody. *J Biol Chem*, **272**, 3302-3308.
8. Baba, K., Tumuraya, K., Tanaka, I., Yao, M. and Uchiumi, T. (2013) Molecular dissection of the silkworm ribosomal stalk complex: the role of multiple copies of the stalk proteins. *Nucleic Acids Res*, **in press**.
9. Bocharov, E.V., Sobol, A.G., Pavlov, K.V., Korzhnev, D.M., Jaravine, V.A., Gudkov, A.T. and Arseniev, A.S. (2004) From structure and dynamics of protein L7/L12 to molecular switching in ribosome. *J. Biol. Chem.*, **279**, 17697-17706.
